# The Draft Genome of Chinese Endemic Species *Phascolosoma esculenta* (Sipuncula, Phascolosomatidae) Reveals the Phylogenetic Position of Sipuncula

**DOI:** 10.3389/fgene.2022.910344

**Published:** 2022-07-22

**Authors:** Shengping Zhong, Xiaowan Ma, Yan Jiang, Ying Qiao, Longyan Zhao, Lianghua Huang, Guoqiang Huang, Yongzhen Zhao, Yonghong Liu, Xiuli Chen

**Affiliations:** ^1^ Institute of Marine Drugs, Guangxi University of Chinese Medicine, Nanning, China; ^2^ Guangxi Engineering Technology Research Center for Marine Aquaculture, Guangxi Institute of Oceanology Co., Ltd., Beihai, China; ^3^ Key Laboratory of Tropical Marine Ecosystem and Bioresource, Fourth Institute of Oceanography, Ministry of Natural Resources, Beihai, China; ^4^ Guangxi Key Laboratory of Aquatic Genetic Breeding and Healthy Aquaculture, Guangxi Academy of Fishery Sciences, Nanning, China

**Keywords:** Phascolosoma esculenta, sipuncula, genome, nanopore, phylogenomics

## Introduction

Lophotrochozoa, the sister taxon of Ecdysozoa, is an ecologically and morphologically diverse clade of protostome animals within the Spiralia (Bleidorn, 2019). Lophotrochozoans represent more than one-third of known marine animals and include the segmented annelids (e.g., Errantia and Sedentaria worms) and the shell bearing molluscs (e.g., oysters and scallops) (Luo et al., 2018). Annelida, also known as the ringed or segmented worms, is a major phylum within the superphylum Lophotrochozoa (Shen et al., 2009). The annelids play important ecological roles in the marine ecosystem, are comprised of more than 21,000 described species, and constitute the dominant benthic macrofauna in all the major oceans (Weigert et al., 2014). Despite their ecological importance, the phylogenetic positions among annelid groups have not been fully resolved and their deep-level evolutionary relationships are still poorly understood (Struck et al., 2011; Bleidorn, 2019). Over the last decade, phylogenomic analyses have suggested that Sipuncula should be included within annelid groups, together with Echiura, even though historically they were considered separate phyla given their lack of segmentation (Dordel et al., 2010; Schulze and Kawauchi, 2021).

Sipuncula (known as peanut or star worms) are a clade of unsegmented, coelomate marine worms, which inhabit marine sediments and occur worldwide from the intertidal zones to the deep sea (Schulze et al., 2005; Lemer et al., 2015). Sipuncula has about 150 species in 17 genera and a fossil record dating back to the Early Cambrian (Huang et al., 2004; Zhong et al., 2020). The fossil evidence indicates that sipunculans may have undergone evolutionary stasis over the last 520 million years (Huang et al., 2004), and the monophyly of Sipuncula taxon has been supported by morphological and molecular data (Schulze et al., 2007; Mwinyi et al., 2009). However, the phylogenetic position of Sipuncula has changed drastically many times over several decades (Dordel et al., 2010; Weigert and Bleidorn, 2016). Previous cladistic analyses based on morphology proposed a close sister taxon relationship between Sipuncula and Mollusca, because of the presence of the “molluscan cross” organization of micromeres during spiral cleavage (Scheltema, 1993). Due to the lack of segmentation and body appendages, Sipuncula were once regarded as their own separate phyla, closely related to Annelida, but not part of Annelida (Rouse and Fauchald, 1997). In contrast to these morphology-based studies, phylogenomic analyses of large molecular datasets, such as transcriptome (Weigert et al., 2014), expressed sequence tags (ESTs) (Struck et al., 2011) and MicroRNAs (Sperling et al., 2009) favor the inclusion of Sipuncula into annelid taxon. The placement of non-segmented Sipuncula within the segmented Annelida taxon implies that patterns of segmentation within annelids have been evolutionarily labile (Struck et al., 2007), and Sipuncula may have secondarily lost segmentation, as is the case with non-segmented Echiura. Although, the placement of Sipuncula within Annelida has been supported by phylogenomic analyses (Weigert and Bleidorn, 2016), it is debated whether Sipuncula is a deeply-nested Annelid or the sister taxon of Annelids (Andrade et al., 2015; Weigert et al., 2016). Therefore, a robust genomic reconstruction of Sipuncula phylogenetic position within Annelida is needed. However, until now, genome data from Sipuncula has not been published.

*Phascolosoma esculenta*, a Sipuncula species restricted to the coastal zone of southeast China, is a valuable fisheries resource for both nutrition and ingredients of Chinese traditional medicines (Zhao et al., 2019). As a burrowing organism thriving in the intertidal zone ecosystem, *P. esculenta* has evolved an extraordinary resilience to harsh and dynamically changing intertidal stresses (Su et al., 2010; Shen et al., 2021). Although, *P. esculenta* has high tolerance of abiotic stress, including temperature, salinity, and hypoxia, the molecular mechanism bolstering *P. esculenta* against intertidal stresses has not been thoroughly investigated (Meng et al., 2022). Hence, decoding the genome of *P. esculenta* will allow for a more thorough understanding of Sipuncula’s ecological adaptation and phylogenetic evolution. In this report, we provided a draft genome of *P. esculenta* using Oxford Nanopore Technologies. We assembled the genome sequences into 1,446 contigs with a total length of 1.71 Gb and a contig N50 length of 2.49 Mb. Furthermore, a total of 1,688 gene families were identified as species-specific of *P. esculenta*, and 1,032 gene families were significantly expanded in the *P. esculenta* genome. The availability of the first genome data from Sipuncula offers better insight into deep-level evolutionary analysis of Annelida, and also provides a valuable resource for the analysis of ecological adaptation and the molecular mechanism of *P. esculenta* against intertidal stresses.

## Materials and Methods

### Sampling, Library Construction, and Sequencing

**FIGURE 1:**
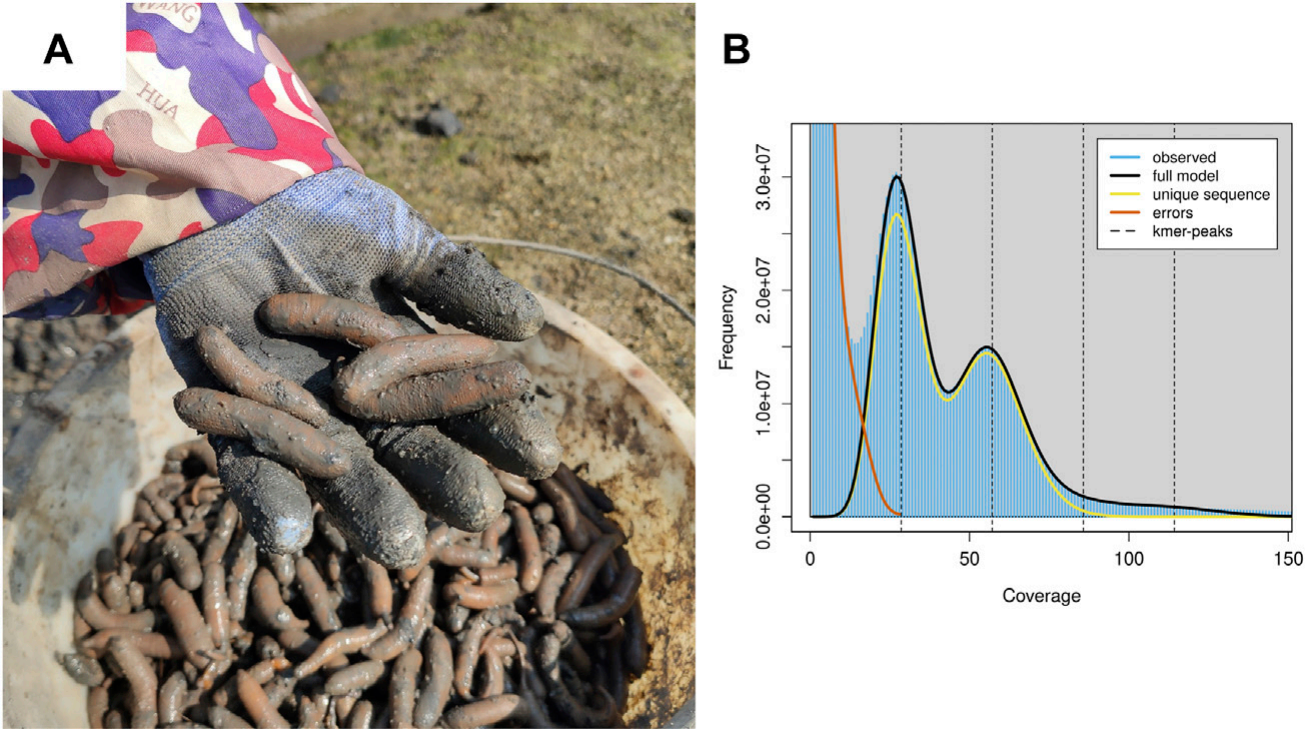
Harvest *P. esculenta* and the genomics feature of *P. esculenta*. **(A)** Harvest *P. esculenta* from local aquaculture farms. **(B)** A K-mer analysis of the genome sequencing reads for the *P. esculenta* using GenomeScope v2.0.

A healthy individual of *P. esculenta* (body weight 4.87 g) was obtained from local aquaculture farms (Figure 1A) (Beihai, Guangxi Province, China, 21.473645 N, 109.469912 E). The introvert, trunk, intestine and nephridia were collected, immediately frozen and stored in liquid nitrogen until extraction of the genomic DNA and total RNA. Total genomic DNA was extracted from the muscle tissue of the trunk sample using the QIAamp DNA Mini Kit (QIAGEN, Hilden, Germany). Approximately 1 µg of genomic DNA was used for constructing the Nanopore 20 kb insert library; the large size fraction of DNA (>20 kb) was selected by automated gel electrophoresis (BluePippin, Sage Science); the sequencing library was prepared by standard ligation sequencing kit (SQK-LSK109, Oxford Nanopore Technologies); and the constructed library was sequenced using the Oxford Nanopore MinION platform at BGI Genomics Co., Ltd., Shenzhen, China. Meanwhile, a paired-end DNA sequencing library with insert size 350 bp was constructed and sequenced using the BGISEQ-500 platform according to the manufacturer’s protocol. Finally, total RNAs were extracted from introvert, intestine and nephridia samples using RNAiso kit (TaKaRa, Dalian, China). The RNA sequencing library with insert size 300 bp was constructed and sequenced (2 × 150 bp paired-end) using the BGI DNBseq platform.

### Genome Size Estimation, Genome Assembly and Polishing

The genomic DNA data sequenced by BGISEQ-500 platform was filtered by fastp v0.23.0 (Chen et al., 2018). The k-mer analysis was conducted to estimate the genome size and heterozygosity of *P. esculenta* using Jellyfish v2.3.0 (Marçais and Kingsford, 2011) and Genomescope v2.0 (Ranallo-Benavidez et al., 2020), with the k-mer length set to 19. The Nanopore long read was corrected and then the preliminarily genome was assembled using NextDenovo v2.5.0 assembler (https://github.com/Nextomics/NextDenovo). The preliminarily genome was polished using NextPolish v1.4.0 software (Hu et al., 2020) to correct base errors caused by Nanopore sequencing. Finally, the redundancy in the polished genome was resolved using Purge Dups v. 1.2.5 (Guan et al., 2020), and the assembly completeness was evaluated by Benchmarking Universal Single-Copy Orthologs (BUSCO) software v5.2.2 (Manni et al., 2021) based on the metazoa_odb10 lineages.

### Genome Annotation

Firstly, the comprehensive transposable elements (TEs) in the genome were detected using EDTA package v2.0.0 (Ou et al., 2019) and the genome were further masked using RepeatMasker v4.1.2 (http://www.repeatmasker.org), based on the TE library generated by the EDTA package. Secondly, a combination of *ab initio*, homology, and transcript-based prediction methods were used to detect the protein-coding genes in the genome. The RNA sequencing data of three tissues were used for transcript-based prediction. *De novo* transcript sequences were reconstructed using the Trinity platform v2.13.2 (Haas et al., 2013), and genome guided transcript sequences were performed using HISAT v2.2.1 and StringTie v2.2.0 (Pertea et al., 2016). A combination of the *de novo* and genome-guided transcript was aligned to the genome by PASA software v2.5.2 (Haas et al., 2008) to obtain transcript-based predicted protein. For the homology-based method, the protein data of Annelida, including *Owenia fusiformis*, *Helobdella robusta*, *Dimorphilus gyrociliatus*, *Lamellibrachia luymsi*, *Capitella teleta*, and *Enchytraeus crypticus* from Genbank were used as reference data to predict the protein-coding genes in the *P. esculenta* genome by GeMoMa program v1.7.1 (Keilwagen et al., 2018). For the *ab initio*-based method, gene prediction was performed using BRAKER2 (Brůna et al., 2021) with protein and RNA-seq forms of evidence. Finally, the gene structures in the *P. esculenta* genome were predicted by a combination of the three methods with EvidenceModeler v1.1.1 (Haas et al., 2008), and the predicted protein-coding genes’ functional annotation was performed using BLASTP v2.12.0 + on a local high performance server (PowerEdge T630, Dell Technologies), with the E-value cutoff of 1e-5, to search the homologous sequences from the public databases, including UniProtKB/Swiss-Prot, UniProtKB/TremBL, Kyoto Encyclopedia of Genes and Genomes (KEGG) and Gene Ontology (GO) protein databases. The gene annotation integrity was evaluated by BUSCO software v5.2.2 based on the metazoa_odb10 lineages.

The transfer RNAs (tRNAs) were identified using tRNAscan-SE v2.0.6 (Chan et al., 2021); microRNAs were identified using miRNAture v1.0 (Velandia-Huerto et al., 2021); and the transfer RNAs (tRNAs) were identified using RNAmmer v1.2 (Lagesen et al., 2007). The other small nuclear RNAs (snRNAs) were identified by searching against the Rfam database using Infernal v1.1.2 (Nawrocki and Eddy, 2013).

### Phylogenomics and Gene Family Evolution

To investigate the phylogenetic status of Sipuncula, the protein data of 16 Lophotrochozoa species including *Mizuhopecten yessoensis*, *Pecten maximus*, *Crassostrea gigas*, *C. virginica*, *Aplysia californica*, *Pomacea canaliculata*, *Gigantopelta aegis*, *Octopus bimaculoides*, *O. sinensis*, *Lingula anatine*, *Phoronis australis*, *C. teleta*, *L. luymsi*, *D. gyrociliatus*, *H.* robusta, and *O. fusiformis* were retrieved from the NCBI genome database (https://www.ncbi.nlm.nih.gov/genome/), and the longest transcript of each gene was selected for identification of the orthologous gene. The orthologous gene clusters of *P. esculenta* and 16 related species were identified using OrthoFinder v2.5.4 (Emms and Kelly, 2019); the single-copy protein sequences were aligned by MUSCLE v3.8.31 with default parameters; and the ambiguously aligned positions were trimmed by trimAl v1.4.1 with default parameters. The alignments of the single-copy sequences were concatenated into continuous super protein sequences, and then the super protein sequences were used to construct the phylogenetic tree using RAxML v8.2.12 (Stamatakis, 2014) under Gamma + LG + F amino acid substitution model with 1,000 bootstrap replicates. Analysis of the likelihood for gene family gain and loss of *P. esculenta* and 16 related species was performed by CAFE v4.2.1 (Han et al., 2013) with *p* < 0.05. Divergence time between Lophotrochozoa species was estimated using r8s v1.71, with the divergence time of *M. yessoensis* and *P. maximu*s obtained from the TimeTree website (http://www.timetree.org) for calibration. The GO and KEGG enrichment analyses were carried out using TBtools (Chen et al., 2020) to analyze the significant expansion in gene family function from *P. esculenta*.

## Results and Discussion

For genome assembly of *P. esculenta*, approximately 81.26 Gb clean Nanopore long reads, with average read length of 20,978 bp and 112.40 Gb clean illumina short reads with 96.11% Q20, were generated (Supplementary Table S1). To estimate the main genome characteristics of *P. esculenta*, the k-mer-based method based on illumina short data was applied. The estimated genome size of *P. esculenta* is about 1,465.56 Mb, the repeat content and the heterozygous rate of the genome were about 57.84 and 2.62%, respectively (Figure 1B, Supplementary Table S2). The estimated genome size is smaller than *Phascolosoma scolops,* which was about 1760.04 Mb by flow cytometer analysis (Adachi et al., 2016).

The initial assembly yielded a total length of 1.99 Gb, comprising 2,206 contigs with a contig N50 length of 2.04 Mb. Due to high heterozygosity of the *P. esculenta* genome, the initial genome assembly was larger than the estimated genome size by k-mer-based method. After correcting base errors of the initial genome assembly, we resolved the redundancy of genome assembly by Purge_Dups. The final genome assembly was 1.71 Gb in total length, comprising 1,446 contigs with a contig N50 of 2.49 Mb, and the largest contig was 12.99 Mb in length (Table 1). The BUSCO analysis was then performed to evaluate the completeness of the final genome assembly, and the result showed that the completeness of this assembled genome was 98.5% (95.0% complete BUSCOs plus 3.5% fragmented BUSCOs) (Supplementary Table S3). The overall genome completeness of *P. esculenta* is one of the highest among the published annelid genomes (Li et al., 2019; Sun et al., 2021), which indicates that the genome integrity is high in the final genome assembly. To further validate the assembly completeness, the BGISEQ-500 short read data were mapped to the final assembly with BWA v0.7.17, and the mapping rate was 94.37%.

**TABLE 1:** Summary statistics of genome assembly and gene prediction of *P. esculenta*.

**Summary Statistics of genome assembly**	
Total length of genome (Gbp)	1.71
Contig N50 size (Mbp)	2.49
Contig number	1,446
The length of largest contig (Mbp)	12.99
Proportion of BUSCO in genome model (%)	98.5
**Summary statistics of gene prediction**	
Protein-coding gene number	41,469
The length of largest protein-coding gene (bp)	38,466
Mean transcript length (bp)	1,270
Mean exons length (bp)	201
Mean exons number per gene	6.3
Proportion of BUSCO in proteins model (%)	99.4

A total of 60.15% of the *P. esculenta* genome (1.03 Gb) were identified as repetitive elements, similar to the estimate in the tubeworm *Paraescarpia echinospica* genome (55.10%) (Sun et al., 2021). The *P. esculenta* genome is among the highest percentage of repetitive sequences among the published annelid genomes, and the most abundant transposable elements were terminal inverted repeats (TIRs, 30.18% of the genome), followed by long terminal repeats (LTRs, 18.95% of the genome) and helitron (5.97%) (Supplementary Table S4). Meanwhile, a total of 41,469 genes in the *P. esculenta* genome were predicted as protein-coding genes by a combination of three gene identifying methods with EvidenceModeler. BUSCO analysis of these protein-coding genes showed that 99.4% of metazoan core conserved genes were detected in the *P. esculenta* gene set, with 98.7% and 0.7% being identified as complete and fragmented, respectively (Supplementary Table S3). Approximately 87.11% of the predicted protein-coding genes were successfully annotated by at least one of the public databases: Swiss-Prot (66.34%), TremBL (87.10%), KEGG (37.13%), and GO (43.56%) (Supplementary Table S5). Furthermore, approximately 0.58 Mb of the genome were annotated as non-coding RNAs, including 1,699 microRNAs, 2,894 tRNAs, 120 rRNAs, and 170 snRNAs (Supplementary Table S6).

To reveal the phylogenetic relationships between *P. esculenta* and other lophotrochozoan species, OrthoFinder was applied for identification of the orthologous genes. A total of 4,445 orthologous genes clusters shared by all species were identified, including 245 single-copy orthologous in a 1:1:1 manner. A total of 1,688 orthogroups (6,971 genes) were identified as species-specific orthogroups of *P. esculenta* (Supplementary Table S7). The phylogenetic tree was constructed with 245 single-copy orthologous genes of 73,344 amino acid sites using RAxML, and the result showed that *P. esculenta* (Sipuncula), *O. fusiformis* (Oweniidae), and other annelid species including *L. luymsi* (Siboglinidae), *C. teleta* (Capitellidae), *D. gyrociliatus* (*Dinophilidae*) and *H. robusta* (Glossiphoniidae) were clustered and constituted the annelid clade. *P. esculenta* (Sipuncula) was found clustered within Annelida, and together with *O. fusiformis* (Oweniidae) taxa, occupied the basal branch of the annelid clade (Supplementary Figure S1). Our phylogenomics analysis result indicated that Sipuncula is in the basal branching position of Annelida, which is congruent with previous phylogenomics studies using mitogenome (Weigert et al., 2016) and transcriptomic (Weigert et al., 2014; Weigert and Bleidorn, 2016) analysis.

**FIGURE 2:**
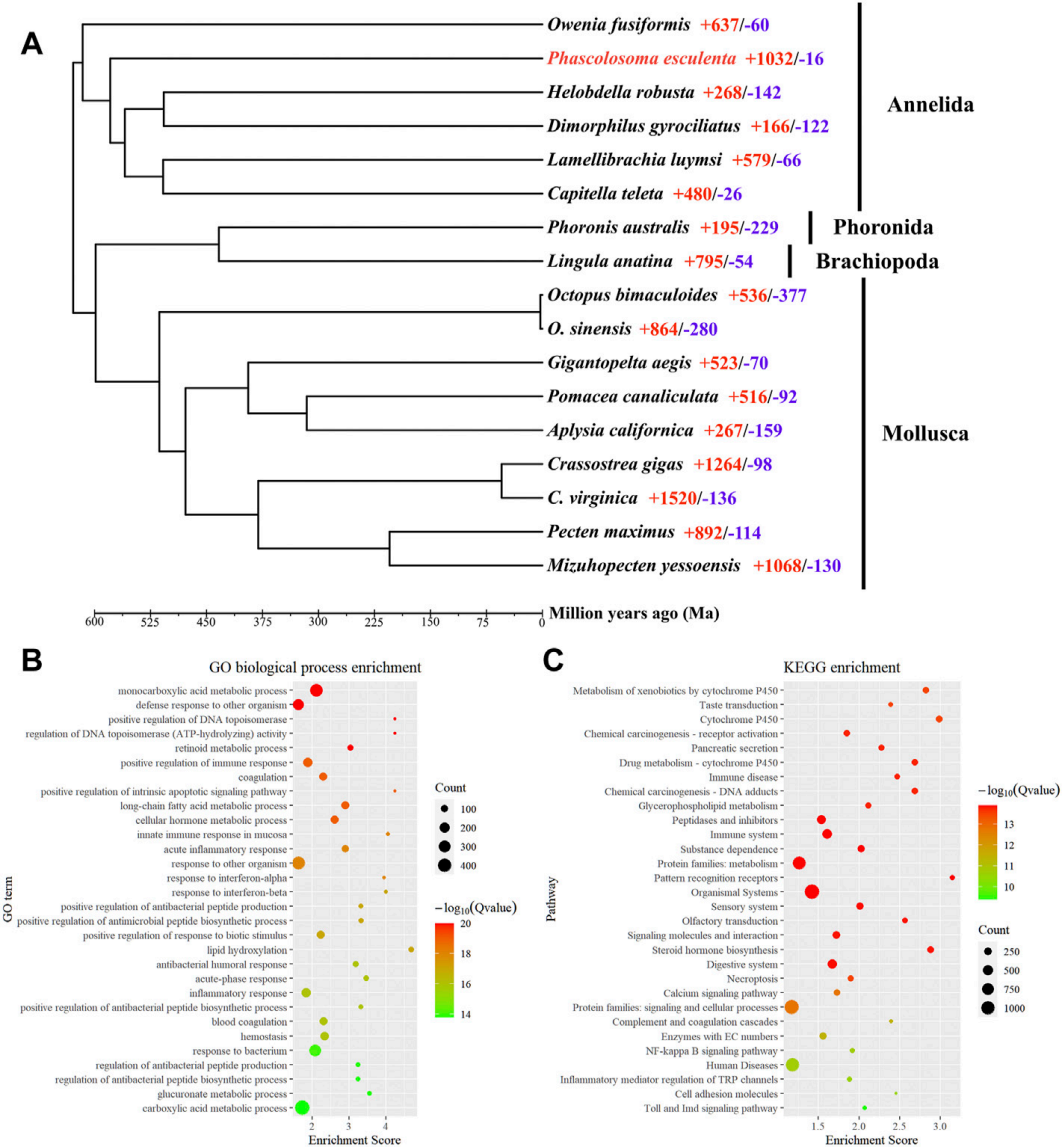
Phylogeny, gene family evolution, and function enrichment analyses. **(A)** Phylogeny and gene family evolution. The number of significantly expanded (red) and contracted (blue) gene families is designated beside the species scientific name. **(B)** Function enrichment of Gene Ontology (GO) for significantly expanded gene families. **(C)** Kyoto Encyclopedia of Genes and Genomes (KEGG) enrichment analysis for significantly expanded gene families. Only the top 30 categories are shown.

Computational analysis of gene family evolution between *P. esculenta* and other lophotrochozoan species revealed that 1,032 and 16 gene families were significantly expanded and contracted in the *P. esculenta* genome, respectively (Figure 2A). Superoxide dismutases (SODs) have an important functional role in protecting cells against oxidative damage induced by environmental stress (Finkel and Holbrook, 2000; Surai et al., 2019). Genomic expansions of Cu/Zn superoxide dismutase (Cu/Zn-SOD) genes and Mn superoxide dismutase (Mn-SOD) in *P. esculenta* genome were revealed by likelihood analysis. Most annelid genomes contain less than three copies of the SOD gene (Sun et al., 2021), but *P. esculenta* genome has 12 copies of the Cu/Zn-SOD gene and 15 copies of the Mn-SOD gene, the largest number of copies among the published annelid genomes. Significant expansion of SODs in the *P. esculenta* genome may play an important role in overcoming oxidative damage induced by intertidal stress and could be essential for *P. esculenta* to inhabit the intertidal zone. Moreover, the GO and KEGG enrichment analyses with 1,032 significantly expanded gene families were performed to clarify the molecular biological function of gene family evolution in *P. esculenta*. GO enrichment analysis revealed that these expanded gene families are mainly involved in defense responses against other organisms (GO:0098542, GO:0051707), positive regulation of immune response (GO:0050778, GO:0002227), blood coagulation (GO:0050817, GO:0007596), positive regulation of the apoptotic signaling pathway (GO:1902231), and antibacterial humoral response (GO:0019731) (Figure 2B). Meanwhile, KEGG enrichment analysis showed that these expanded gene families were significantly enriched in 91 pathways, including immune and defense associated pathways, such as metabolism of xenobiotics by cytochrome P450, the immune system, pattern recognition receptors, complement and coagulation cascades, and the Toll and Imd signaling pathway (Figure 2C). Several gene families related to defense pathways—including immune responses, apoptosis and anti-oxidation—were also expanded in the *C. gigas* genome. The expansion of key immune and defense genes indicates the sophisticated genomic adaptations of the oyster in inhabiting a highly stressful environment (Zhang et al., 2012). The defense related gene family cytochrome P450 (CYPs) were also significantly expanded in the *Daphnia pulex* genome, which provides important insight into the adaptation of *D. pulex* to environmental changes (Baldwin et al., 2009). So too the GO and KEGG enrichment analysis results in this study suggest that genomic expansions of immune and defense associated gene families offer important insight into the ecological adaptation of *P. esculenta* to environmental stresses in the intertidal zone.

## Data Availability

The datasets presented in this study can be found in online repositories. The names of the repository/repositories and accession number(s) can be found in the article/Supplementary Material.
