# Supplementary material for: The Draft Genome of Chinese Endemic Species Phascolosoma esculenta (Sipuncula, Phascolosomatidae) Reveals the Phylogenetic Position of Sipuncula
Source: Front Genet. 2022 Jul 22;13:910344. doi: 10.3389/fgene.2022.910344 (PMC9354978; doi:10.3389/fgene.2022.910344)
Supplement: Supplementary file 1 [file DataSheet2.DOCX]

**Supplementary Table 1.** Summary of obtained sequencing data generated for *P. esculenta* genome assembly and gene prediction.

| **Sequencing libraries** | **Insert size** | **Clean data (Gb)** | **Read Length (bp)** |
| --- | --- | --- | --- |
| Nanopore | 20 k | 81.26 | 20,978(Average) |
| BGISEQ-500 | 350 bp | 112.40 | 150 |
| RNA-seq | 300 bp | 60.70 | 150 |
| Total | - | 254.36 | - |

**Supplementary Table 2.** Estimation of genome size based on 19-mer statistics.

| Kmer | Depth | Genome size (Mb) | repeat content (%) | Heterozygous rate (%) |
| --- | --- | --- | --- | --- |
| 19 | 28.60 | 1465.55 | 57.84% | 2.62 |

**Supplementary Table 3.** The BUSCO result of *P. esculenta* genome assembly and gene prediction.

|  | Term | Number | Ratio (%) |
| --- | --- | --- | --- |
| genome assembly | Complete BUSCOS (C) | 906 | 95.0 |
|  | Single-copy BUSCOS (S) | 861 | 90.3 |
|  | Duplicated BUSCOS (D) | 45 | 4.7 |
|  | Fragmented BUSCOS (F) | 33 | 3.5 |
|  | Missing BUSCOS (M) | 15 | 1.5 |
| gene prediction | Complete BUSCOS (C) | 941 | 98.7 |
|  | Single-copy BUSCOS (S) | 904 | 94.8 |
|  | Duplicated BUSCOS (D) | 37 | 3.9 |
|  | Fragmented BUSCOS (F) | 7 | 0.7 |
|  | Missing BUSCOS (M) | 6 | 0.6 |

**Supplementary Table 4.** Summary statistics for the annotated repeat sequences.

| Repeat Classes | Count | Length (bp) | % of genome |
| --- | --- | --- | --- |
| terminal inverted repeats | 634146 | 323939037 | 0.1895 |
| long terminal repeats | 1631940 | 515813302 | 0.3018 |
| non-long terminal repeats | 15471 | 8610942 | 0.0050 |
| helitron | 286204 | 102051592 | 0.0597 |
| tandem repeats | 202113 | 77697830 | 0.0455 |
| Total | 2769874 | 1028112703 | 0.6015 |

**Supplementary Table 5.** Statistics for the functional annotation of protein-coding genes.

| **Database** | **Gene Number** | **Percent (%)** |
| --- | --- | --- |
| Swiss-Prot | 27,511 | 66.34 |
| TremBL | 36,121 | 87.10 |
| KEGG | 15,398 | 37.13 |
| GO | 18,063 | 43.56 |
| At least one database | 36,123 | 87.11 |
| Total | 41,469 | - |

**Supplementary Table 6.** Summary statistics of non-coding RNA annotation.

|  | **Type** | **Number** | **Average Length(bp)** | **Total Length(bp)** | **%Genome** |
| --- | --- | --- | --- | --- | --- |
| microRNAs | - | 1,699 | 84 | 142,857 | 0.0084 |
| tRNAs | - | 2,894 | 77 | 225,651 | 0.0132 |
| rRNAs | 5S | 53 | 116 | 6,182 | 0.0004 |
|  | 5.8S | 11 | 149 | 1,644 | 0.0001 |
|  | 18S | 31 | 1615 | 50,086 | 0.0029 |
|  | 28S | 25 | 5084 | 127,112 | 0.0074 |
|  | Total | 120 | 1541 | 185,024 | 0.0108 |
| snRNAs | CD-box | 31 | 90 | 2,814 | 0.0002 |
|  | HACA-box | 8 | 216 | 1,728 | 0.0001 |
|  | splicing | 131 | 163 | 21,450 | 0.0013 |
|  | Total | 170 | 152 | 25,992 | 0.0015 |

**Supplementary Table 7.** Summary statistics for orthogroups in *P. esculent*a genome.

|  | *P. esculenta* |
| --- | --- |
| Number of genes | 41469 |
| Number of genes in orthogroups | 35910 |
| Number of unassigned genes | 5559 |
| Percentage of genes in orthogroups (%) | 86.6 |
| Percentage of unassigned genes (%) | 13.4 |
| Number of orthogroups containing species | 13764 |
| Percentage of orthogroups containing species (%) | 37.4 |
| Number of species-specific orthogroups | 1688 |
| Number of genes in species-specific orthogroups | 6971 |
| Percentage of genes in species-specific orthogroups (%) | 16.8 |
