# Supplementary material for: The Draft Genome of Chinese Endemic Species Phascolosoma esculenta (Sipuncula, Phascolosomatidae) Reveals the Phylogenetic Position of Sipuncula
Source: Front Genet. 2022 Jul 22;13:910344. doi: 10.3389/fgene.2022.910344 (PMC9354978; doi:10.3389/fgene.2022.910344)
Supplement: Supplementary file 2 [file DataSheet1.DOCX]

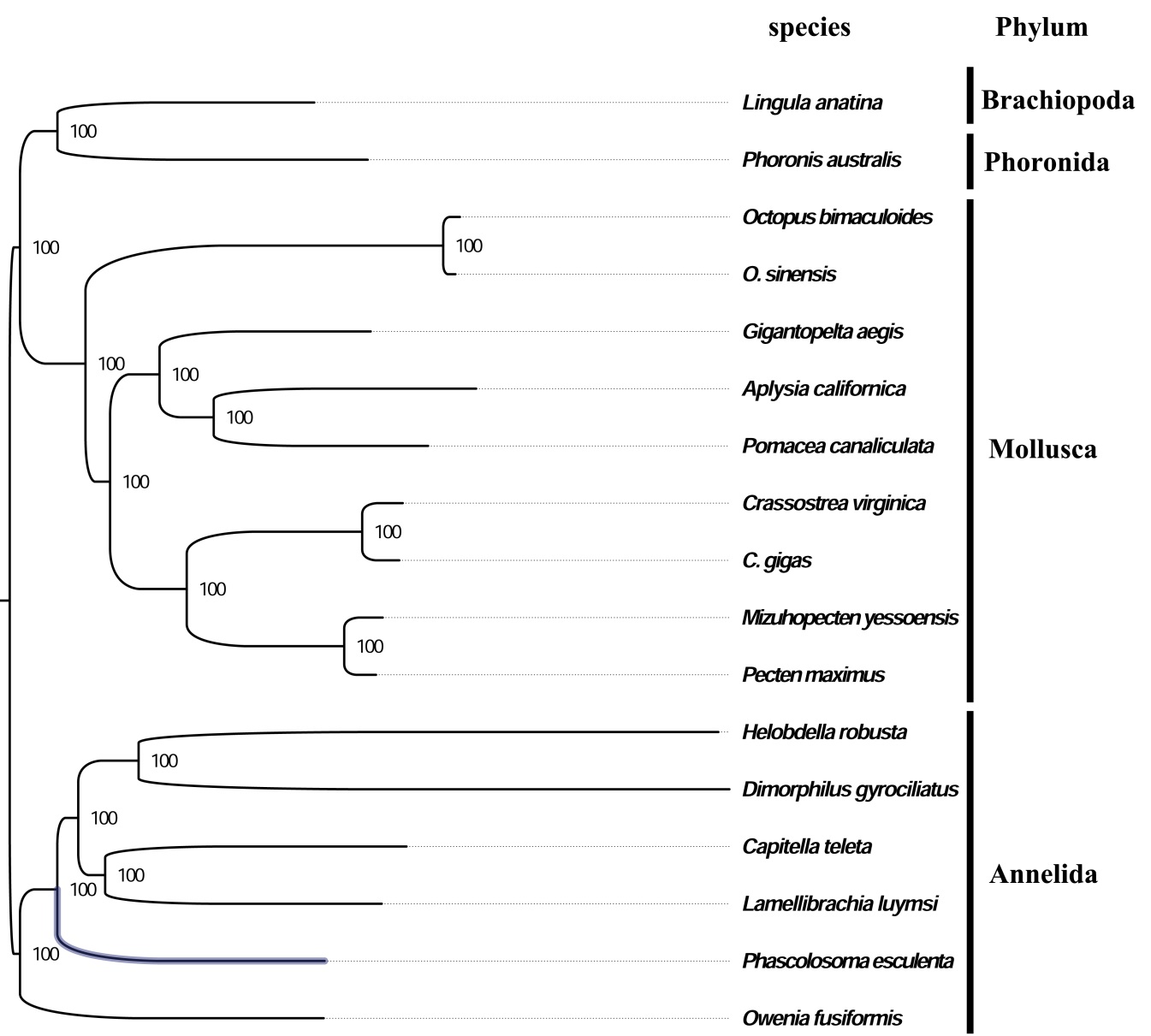


Supplementary Figure 1. Phylogenetic relationships between *P. esculenta* and other lophotrochozoan species constructed with 245 single-copy orthologous using RAxML, the bootstrap values were labeled at each branch nodes .
